# Supplementary material for: Dupilumab Improves Skin Barrier Function in Adults with Atopic Dermatitis: A Prospective Observational Study
Source: J Clin Med. 2022 Jun 10;11(12):3341. doi: 10.3390/jcm11123341 (PMC9225017; doi:10.3390/jcm11123341)
Supplement: Supplementary file 1 [file jcm-11-03341-s001.zip › jcm-1732016-supplementary.pdf]

Supplementary Materials

Table S1. Changes in clinical scores between groups.

	**Topical corticosteroids (*n* = 10)**				**Cyclosporine (*n* = 14)**				**Dupilumab (*n* = 22)**				***P*-value#**
	Baseline (n = 10)	End (n = 9)	Change (n = 9)	***P*-value***	Baseline (n = 14)	End (n = 12)	Change (n = 12)	***P*-value***	Baseline (n = 22)	End (n = 21)	Change (n = 21)	***P*-value***	
**EASI**	11.72 (3.91)	4.54 (2.20)	-7.05	*0.008*	15.04 (4.09)	11.21 (8.67)	-5.14	*0.092*	24.60 (5.36)	7.07 (4.33)	-17.50	*<0.001*	*<0.001*
**SCORAD**	37.28 (12.11)	24.33 (13.15)	-12.79	*0.073*	47.41 (9.43)	32.93 (20.64)	-15.69	*0.03*	57.30 (13.83)	27.29 (10.38)	-29.26	*<0.001*	*0.026*
**DLQI**	12.20 (6.20)	9.38 (5.01)	-3.75	*0.195*	9.29 (4.05)	6.11 (4.57)	-2.56	*0.196*	17.59 (7.28)	5.19 (4.62)	-12.52	*<0.001*	*0.002*
**BSA**	14.23 (5.31)	7.68 (4.51)	-6.33	*0.006*	22.63 (7.96)	19.26 (17.39)	-6.61	*0.234*	39.54 (18.47)	17.18 (12.24)	-21.94	*<0.001*	*0.012*
**IGA**	2.50 (0.53)	1.25 (0.46)	-1.13	*0.002*	3.21 (0.58)	2.33 (1.11)	-1.00	*0.028*	3.73 (0.46)	1.90 (0.70)	-1.81	*<0.001*	*0.022*

Data are expressed as mean (standard deviation).

BSA, body surface area; DLQI, Dermatology Life Quality Index; EASI, Eczema Area Severity Index; IGA, Investigator Global Assessment scale; SCORAD, SCORing Atopic Dermatitis.

**P-value* after using Student's t-test for paired samples to compare scores at baseline and after follow-up.

#*P-value* after using one-way independent ANOVA test to compare scores between different treatments (topical corticosteroids, cyclosporine and dupilumab).

Table S2. Changes in barrier function parameters after each treatment and between groups.

**Skin homeostasis parameters**	**Topical corticosteroids (n = 10)**				**Cyclosporine (n = 14)**				**Dupilumab (n = 22)**				**Differences between treatments**
	Baseline (n = 10)	End (n = 9)	Change (n = 9)	***P-value****	Baseline (n = 14)	End (n = 12)	Change (n = 12)	***P-value****	Baseline (n = 22)	End (n = 21)	Change (n = 21)	***P-value****	***P-value#***
**On eczematous lesions**													
**TEWL ($g·h^{-1}·m^{-2}$)**	25.30 (10.59)	14.88 (3.41)	-10.43	0.047	23.51 (9.88)	15.79 (6.75)	-7.73	0.089	31.02 (16.93)	12.10 (5.05)	-18.93	<0.001	0.128
**Temperature (ºC)**	31.54 (1.46)	32 (1.06)	+0.46	0.257	32.35 (1.13)	32.74 (046)	+0.49	0.316	32.53 (1.08)	31.64 (1.56)	-0.82	0.009	0.013
**SCH (AU)**	18.24 (12.53)	40.79 (13.86)	+22.54	0.010	27.78 (15.05)	34.36 (17.79)	+2.85	0.986	19.93 (9.71)	37.73 (15.59)	+19.18	<0.001	0.029
**pH**	6.82 (0.50)	6.77 (0.57)	-0.04	0.902	6.12 (0.52)	6.37 (0.68)	+0.86	0.064	6.56 (0.80)	6.45 (0.67)	-0.12	0.641	0.177
**On non-lesioned skin**													
**TEWL ($g·h^{-1}·m^{-2}$)**	9.35 (3.06)	9.49 (3.92)	+0.14	0.940	10.83 (4.54)	9.23 (2.69)	-1.60	0.257	11.87 (4.77)	8.25 (2.15)	-3.62	0.006	0.159
**Temperature (ºC)**	30.83 (1.58)	31.26 (0.99)	+0.44	0.227	31.25 (1.25)	31.70 (0.84)	+0.61	0.306	31.18 (1.28)	30.47 (1.48)	-0.63	0.094	0.083
**SCH (AU)**	39.94 (9.21)	46.40 (10.61)	+6.46	0.061	38.23 (12.85)	39.09 (11.46)	+0.05	0.620	32.68 (12.59)	41.68 (10.63)	+11.41	<0.001	0.033
**pH**	6.34 (0.66)	6.77 (0.89)	+1.81	0.421	6.07 (0.54)	6.49 (0.65)	+0.99	0.021	6.57 (0.76)	6.58 (0.65)	-0.02	0.953	0.173

Data are expressed as mean (standard deviation).

AU, arbitrary units; SCH, Stratum Corneum Hydration; TEWL, Transepidermal Water Loss.

**P-value* after using Student's t-test for paired samples to compare parameters at baseline and after follow-up.

#*P-value* after using the one-way independent ANOVA test to compare changes in skin homeostasis parameters between different treatments (topical corticosteroids, cyclosporine and dupilumab

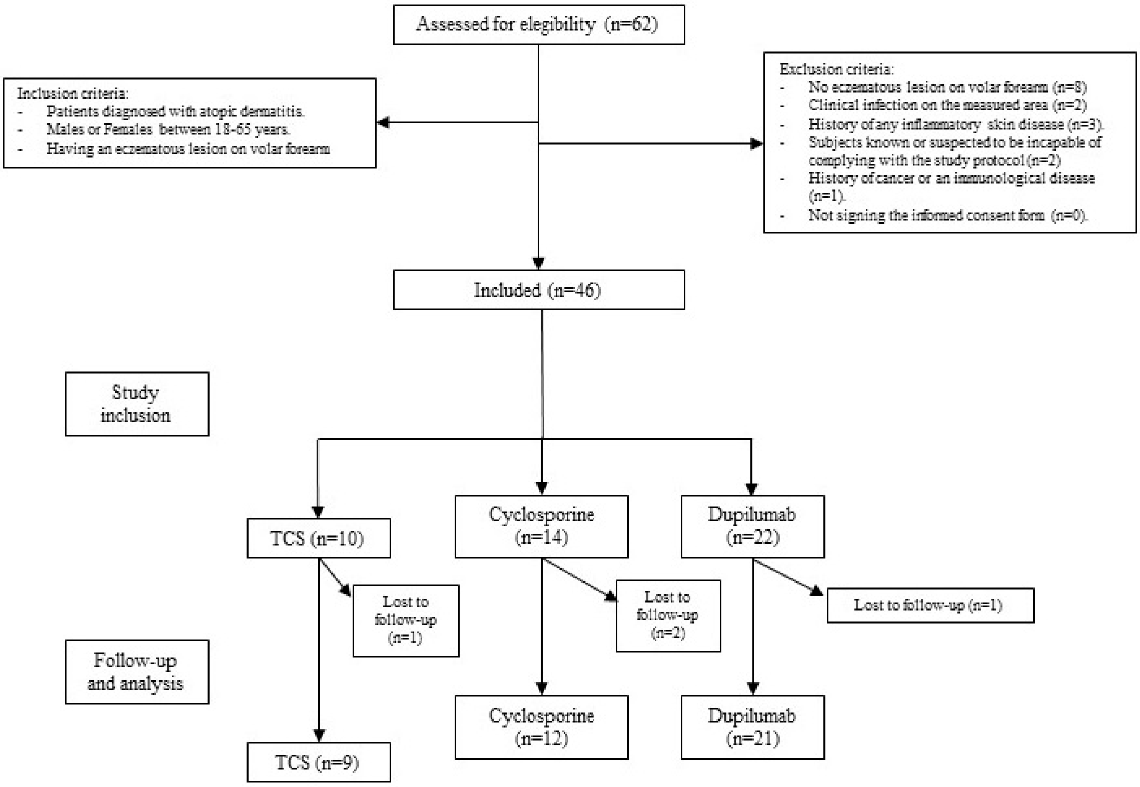

Figure S1. Participant flow chart
